# Sleep, activity, and diet in harmony: unveiling the relationships of chronotype, sleep quality, physical activity, and dietary intake

**DOI:** 10.3389/fnut.2023.1301818

**Published:** 2023-12-15

**Authors:** Ahmet Murat Günal

**Affiliations:** Faculty of Health Sciences, Department of Nutrition and Dietetics, İstanbul Okan University, İstanbul, Türkiye

**Keywords:** chrononutrition, chronotypes, sleep quality, physical activity, dietary intake, nutrition

## Abstract

**Introduction:**

This cross-sectional study aims to explore the intricate relationships among chronotype, sleep quality, physical activity, and dietary intake in a diverse cohort of 3,072 (50.2% female) participants residing in İstanbul, Türkiye.

**Methods:**

This study utilized established measurement tools, including the Morningness-Eveningness Questionnaire (MEQ) to assess chronotype, the Pittsburgh Sleep Quality Index (PSQI) to evaluate sleep quality, the International Physical Activity Questionnaire Short Form (IPAQ-SF) to measure physical activity, and a 24-h dietary recall method to assess dietary intake.

**Results:**

The findings of this study revealed compelling associations. Firstly, a robust association was observed between sleep quality and chronotype (OR: 2.265; 95% CI: 1.954–2.626; *p* < 0.001) as well as physical activity (OR: 0.836; 95% CI: 0.750–0.932; *p* = 0.002). Specifically, evening chronotypes are more likely to have poor sleep quality, while highly active individuals tend to report lower sleep quality. Transitioning from inactivity to high activity was associated with a 16.4% increase in the odds of transitioning from normal to poor sleep, while a shift from an evening to a morning chronotype was linked to a substantial 126.5-fold increase in the odds of moving from poor to normal sleep. Additionally, morning chronotypes also display distinctive dietary patterns, characterized by higher energy, protein, and fat intake, and reduced carbohydrate intake. Poor sleep quality is associated with increased energy and macronutrient consumption.

**Discussion:**

These findings underscore the intricate relationships of chrononutrition within the context of sleep quality, physical activity, and dietary choices. The study underscores the significance of personalized interventions to effectively address specific health behaviors, highlighting the complexity of chrononutrition’s role in promoting overall health and wellbeing.

## 1 Introduction

The intricate interplay between sleep, physical activity, and dietary intake has garnered substantial attention in the realm of health sciences, underscored by the recognition of their collective influence on individual wellbeing. Emerging research has highlighted the significance of sleep duration and quality in shaping various aspects of human physiology and metabolism, while parallel investigations have illuminated the profound impact of dietary choices and physical activity levels on overall health outcomes. Furthermore, the recognition of chronotype has added a nuanced layer to the complex relationship between these factors (1–4).

Chronotype refers to an individual’s inherent preference for either morning or evening activities, stemming from the interplay between biological rhythms and external societal schedules. It signifies when an individual is most alert, productive, and responsive to environmental cues. Morning chronotypes, often termed “morning people,” experience heightened alertness and cognitive function during the early hours of the day, while evening chronotypes, colloquially referred to as “night owls,” are more active and attentive during later hours. The distinction between these chronotypes is influenced by the social environment, genetic predisposition, and hormonal fluctuations that regulate the sleep-wake cycle (5).

Sleep quality encapsulates various dimensions of sleep experiences beyond the duration of sleep. It is defined by the subjective satisfaction and restorative nature of sleep. Sleep quality encompasses factors such as sleep latency (time taken to fall asleep), sleep efficiency (ratio of time asleep to time spent in bed), sleep disturbances (frequency of awakenings), sleep duration (total time slept), use of sleep medication, daytime dysfunction (impact of sleep problems on daytime functioning), and overall sleep satisfaction. Optimal sleep quality is characterized by uninterrupted and restful sleep, while poor sleep quality is associated with frequent awakenings, discomfort, and daytime impairment (6).

The interplay between chronotype, sleep quality, physical activity, and dietary intake underscores the intricate nature of human health and wellbeing. The chronotype influences an individual’s sleep-wake patterns, activity preferences, and alertness levels throughout the day. Sleep quality, in turn, is influenced by the alignment of sleep patterns with individual chronotypes, impacting overall restfulness and vitality (7, 8). Physical activity levels, regardless of chronotype, contribute to sleep quality by promoting better sleep architecture and reducing sleep disturbances (3, 9). Similarly, dietary intake, influenced by both chronotype and physical activity, can also impact sleep quality (10, 11). Conversely, sleep quality can influence dietary choices and appetite regulation (12–14). The multidirectional interactions among these factors create a complex web of influences on health outcomes. Understanding these relationships has implications for tailored interventions that optimize sleep, physical activity, and dietary behaviors to enhance overall wellbeing.

The present study aims to unveil the intricate interplay of chronotype, sleep quality, physical activity, and dietary intake within a large participant cohort. By adopting a multifaceted approach, this investigation aims to contribute substantively to the evolving body of knowledge elucidating the relationships between these pivotal domains. In doing so, it aspires to inform targeted interventions that promote healthful synergies between these concepts, thereby fostering a holistic framework for individual wellbeing.

## 2 Materials and methods

### 2.1 Design

This study employed a cross-sectional design, in accordance with the STROBE guidelines (15), conducted between September 2022 and July 2023, following ethical approval. Thirty senior students from the Nutrition and Dietetics Department were purposively selected for data collection. The author provided comprehensive training to ensure accurate data collection, particularly in 24-h dietary recall records. The students volunteered for this study as an extracurricular activity. This approach aimed to maintain data quality and was conducted by established research practices in nutrition and dietetics.

### 2.2 Participants and procedure

Before participation in the study, written informed consent was obtained from all participants. The inclusion criteria included residence in İstanbul/Türkiye and an age range of 18–65 years. Additionally, those who use prescription drugs and have been diagnosed with physiological or psychological diseases that may affect their sleep patterns were not included in the study. Ethical approval was granted by the Ethics Committee of İstanbul Okan University (Date: 12.09.2022; Number: 153-7) under the Helsinki Declaration.

This study’s sample size was determined to accommodate the multifaceted nature of investigating chronotype, sleep quality, physical activity, and dietary intake while aiming to provide statistically robust findings. Complex interactions among these variables necessitated a sizeable cohort to sufficiently capture the diversity of behaviors within the population under investigation. A total of 3,072 individuals (50.2% female) were participated in the study. This allowed for a comprehensive exploration of the intricate relationships between chrononutrition variables, ensuring adequate statistical power to detect meaningful associations while maintaining a balance between feasibility and representativeness. Data collection was conducted through face-to-face interviews, ensuring no exclusions due to incomplete forms.

Participants were randomly recruited on a voluntary basis in multiple city centers of İstanbul. This approach might limit the study’s representation of rural areas or individuals less accessible through city-centered recruitment. But, according to the Turkish Statistical Institute’s (TurkStat) report released in May 2023, only 0.9% of the population of İstanbul resides in rural areas (16). Regarding potential selection bias due to self-selection, it is essential to acknowledge that the voluntary nature of participation might introduce a degree of bias. While the sample’s voluntary nature could influence the generalizability of findings, the diverse demographics and geographical representation of İstanbul/Türkiye within the sample enhance the study’s external validity.

### 2.3 Measurements

The data collection tool consists of an information form, the Morningness-Eveningness Questionnaire (MEQ), the Pittsburgh Sleep Quality Index (PSQI), the International Physical Activity Questionnaire Short Form (IPAQ-SF), and lastly, a 24-h Dietary Recall, respectively.

#### 2.3.1 Information form

The information form comprises a set of inquiries encompassing participants’ anthropometric attributes, including weight and height, along with sociodemographic details such as age, education, and income level. Body mass index (BMI) values were derived from participants’ self-reported weight and height statements and calculated using the formula: BMI = weight(kg)/[height(m)]^2^.

#### 2.3.2 Morningness-Eveningness Questionnaire

The MEQ, developed by Horne and Ostberg (5), represents a pivotal instrument utilized for discerning individuals’ circadian preferences. It was subsequently adapted into Turkish by Pündük et al. (17) and Agargun et al. (18). These adaptations demonstrated high reliability in Turkish samples, with Cronbach’s α coefficients of 0.812 and 0.81, respectively.

The MEQ consists of 19 items, designed to probe respondents’ time preferences in various daily activities, with a total score ranging from 16 to 86 points. Higher scores indicate an inclination toward morningness chronotype. Interpretation categorizes scores into three types: evening type (16–41), neither/intermediate type (42–58), and morning type (59–86).

#### 2.3.3 Pittsburgh Sleep Quality Index

The evaluation of sleep quality was carried out using the Pittsburgh Sleep Quality Index (PSQI) developed by Buysse et al. (6), a self-administered questionnaire designed to evaluate sleep quality over the past month. Turkish adaptation, assessed by Ağargün et al. (19), showed high internal consistency with a Cronbach’s α coefficient of 0.804.

The PSQI encompasses 19 self-rated questions that collectively yield seven distinct components: subjective sleep quality, sleep latency, sleep duration, sleep efficiency, sleep disturbance, use of hypnotic medications, and daytime dysfunction. Each component is scored on a scale ranging from 0 to 3, thereby generating a global PSQI score spanning from 0 to 21. A higher score indicates a lower quality of sleep, with a global PSQI score exceeding 5 categorizes individuals as poor sleepers, experiencing severe difficulties in at least two or moderate difficulties in more than three areas.

#### 2.3.4 International physical activity questionnaire–short form

The IPAQ-SF, collaboratively developed by an international research consortium led by Craig et al. (20), serves as a valuable tool for assessing physical activity patterns in large-scale studies. Its concise format and standardized scoring facilitate insights into population-level physical activity. The Turkish adaptation, executed by Saglam et al. (21), maintains the questionnaire’s effectiveness. The short form evaluates physical activities of at least 10 min duration over the past seven days in terms of frequency, duration (in minutes), and intensity, quantified as the metabolic equivalent of task (MET) value. One MET represents the oxygen consumption of a person at rest (3.5 ml O_2_/kg/min).

The questionnaire comprises four sections: vigorous physical activities, moderate-intensity physical activities, walking, and sitting. In the IPAQ framework, “vigorous physical activities” correspond to an expenditure of 8.0 MET, “moderate-intensity physical activities” equate to 4.0 MET, and “walking” translates to 3.3 MET. To calculate the total MET score, the MET values for specific activities are multiplied by their respective durations and frequencies (days) and then summed. An individual’s overall physical activity level is determined by this score, with a MET score below 600 indicating inactivity, 600–1,500 signifying minimal activity, and a score above 1,500 denoting an active lifestyle.

#### 2.3.5 24-h dietary recall

The final pillar of measurement is dietary intake, assessed through 24-h dietary recall. This method offers a comprehensive means to capture detailed information regarding food and beverage consumption within a specific day. This structured interview process involves collecting data on all items consumed in the past 24 h, typically from midnight to midnight on the previous day (22). The energy and macronutrient consumption of the participants were analyzed using the Turkish Nutrition Information System (BeBiS) program version 8.0. Although the 24-h dietary recall method is well-established and widely used in nutrition research, it is important to note dietary preferences may change from day to day and this approach provides a snapshot of dietary consumption.

### 2.4 Data analysis

In the analysis of the data, SPSS 22.0 and Microsoft Excel 16 software programs were utilized. The criterion for assessing normality was based on the skewness and kurtosis values that fall within the range of ± 1.00. *T*-tests and Mann–Whitney U tests were used for pairwise comparisons in independent samples. One-way analysis of variance and the Kruskal–Wallis H test were applied for comparisons involving three or more groups. The assumption of homoscedasticity was verified using the Levene test. *Post-hoc* analyses were conducted using the Tukey Honestly Significant Difference (HSD), Games-Howell, and Dunn tests. Relationships between categorical variables were assessed using Pearson’s chi-square test. Logistic regression and multinomial logistic regression were employed to determine the effects. All evaluations were carried out with a confidence interval of 95%.

## 3 Results

The study included a total of 3,072 participants, 1,543 (50.2%) of whom were female. In terms of age groups, 1,701 (55.4%) were 25 years and older, while 1,371 (44.6%) were in the 18–24 age range. Regarding educational background, 1,556 (50.7%) had a bachelor’s degree, and 2,033 (66.2%) were single. Concerning income levels, 1,517 (49.4%) reported income equal to expenses. Smoking was reported in 1,806 (58.8%), and 2,431 (79.1%) did not consume alcohol. The participants’ ages ranged from 18 to 65 years, with a mean age of 30.16 and a standard deviation of 10.92 (Table 1).

**TABLE 1:** Participants’ characteristics.

	*n*		%	
**Sex**				
Female	1,543		50.2	
Male	1,529		49.8	
**Age groups**				
18–24 years	1,371		44.6	
25 years and above	1,701		55.4	
**Education status**				
Primary school	122		4	
High school	676		22	
Vocational school	493		16	
Undergraduate	1,556		50.7	
Graduate	225		7.3	
**Marital status**				
Single	2,033		66.2	
Married	1,039		33.8	
**Income status**				
Income < Expense	590		19.2	
Income = Expense	1,517		49.4	
Income > Expense	965		31.4	
**Smoking status**				
No	1,806		58.8	
Yes	1,266		41.2	
**Alcohol consumption**				
No	2,431		79.1	
Yes	641		20.9	
**Total**	3,072		100.0	
	**Min**	**Max**	**Mean**	**SD**
**Age**	18.00	65.00	30.16	10.92

When comparing females and males, significant differences were observed in height, weight, and BMI in favor of males (all *p* < 0.001). In terms of BMI classifications, significant differences were observed as well (*p* < 0.001). Among females, 10.6% were classified as underweight, 64.1% as normal weight, 19.2% as overweight, and 6.1% as with obesity. Among males, 1.3% were underweight, 48% were normal weight, 41.7% were overweight, and 9.0% were with obesity (Table 2).

**TABLE 2:** Participants’ anthropometrics.

	Female		Male		Total		*p*
	Min–max	Mean ± SD	Min–max	Mean ± SD	Min–max	Mean ± SD	
Height (cm)	150–185	164.7 ± 6.1	152–206	178.7 ± 7.1	150–206	171.67 ± 9.63	< 0.001*_a_
Weight (kg)	38–125	62.1 ± 11.4	49–150	81 ± 12.6	38–150	71.50 ± 15.31	< 0.001*_a_
BMI (kg/m^2^)	15.60–44.3	22.9 ± 4.2	15.40–46.3	25.4 ± 3.6	15.43–46.3	24.12 ± 4.1	< 0.001*_a_
	***n***	**%**	***n***	**%**	***n***	**%**	***p***
Underweight (< 18.5 BMI)	163	10.6	20	1.3	183	6.0	< 0.001*_b_
Normal (18.5–24.99 BMI)	989	64.1	734	48.0	1,723	56.1	
Overweight (25.00–29.99 BMI)	297	19.2	637	41.7	934	30.4	
Obesity (≥ 30 BMI)	94	6.1	138	9.0	232	7.6	

^*a*^Independent samples *t*-test.

^*b*^Pearson chi-square.

*p < 0.001.

The participants exhibited a wide range of scores, with an average Chronotype score of 50.01 ± 8.25, an average PSQI score of 6.38 ± 3.02, and an average MET score of 1,647.90 ± 1,203.26. In terms of chronotype, 14.1% were classified as evening type, 72.6% as intermediate type, and 13.2% as morning type. Regarding sleep quality, 56.3% experienced poor sleep quality, while 43.7% had normal sleep quality. Considering physical activity, 11.9% were inactive, 46.3% were minimally active, and 41.8% were highly active (Table 3).

**TABLE 3:** Participants’ scores from scales and distribution into groups.

	Mean	SD
MEQ scores	50.01	8.25
PSQI scores	6.38	3.02
MET scores	1,647.90	1,203.26
	***n***	**%**
**Chronotype**		
Evening type (16–41)	434	14.1
Intermediate type (42–58)	2,231	72.6
Morning type (59–86)	407	13.2
**Sleep quality**		
Poor Sleep Quality (> 5)	1,730	56.3
Normal Sleep Quality (≤ 5)	1,342	43.7
**Physical activity**		
Inactive (< 600)	367	11.9
Minimally Active (600–1,500)	1,422	46.3
Highly Active (> 1500)	1,283	41.8
**Total**	3,072	100.0

There were significant differences in the chronotype, sleep quality, and MET groups of the participants in their various demographic and lifestyle factors. In terms of chronotype, there were significant differences by age group, with the 18–24 age range being more likely to be of the evening type (18.5%) compared to those aged 25 and older (10.6%) (*p* < 0.001). Those who had a primary education were more likely to be morning types (29.5%) compared to those with higher levels of education (*p* < 0.001). Furthermore, there were significant differences in chronotype by marital status, with single individuals more likely to be evening types (16.6%) compared to married ones (9.2%) (*p* < 0.001). For sleep quality, there were significant differences by sex, with 60.1% of females being poor sleepers compared to 52.5% of males (*p* < 0.001). By age group, those aged between 18–24 were less likely to have normal sleep quality (40.6%) compared to those aged 25 and older (46.1%) (*p* = 0.002). Furthermore, those having a higher education level were more likely to report poor sleep quality (*p* = 0.001). There were significant differences in MET groups by sex, with 37.7% of females being highly active compared to 45.8% of males (*p* < 0.001). There were also significant differences in MET groups by smoking status and alcohol consumption, with smokers and drinkers being more likely to be highly active (*p* < 0.001; *p* = 0.001, respectively). Other correlations can be seen in Table 4.

**TABLE 4:** Comparison of sociodemographic characteristics of participants according to chronotype, sleep quality, and physical activity.

	Chronotype							Sleep quality					Physical activity						
	Evening type		Intermediate type		Morning type		*p*	Poor		Normal		*p*	Inactive		Minimally active		Highly active		*p*
	*n*	%	*n*	%	*n*	%		*n*	%	*n*	%		*n*	%	*n*	%	*n*	%	
**Sex**																			
Female	220	14.3	1,125	72.9	198	12.8	0.787	928	60.1	615	39.9	< 0.001***	166	10.8	795	51.5	582	37.7	< 0.001***
Male	214	14.0	1,106	72.3	209	13.7		802	52.5	727	47.5		201	13.1	627	41.0	701	45.8	
**Age groups**																			
18–24 years	253	18.5	1,019	74.3	99	7.2	< 0.001***	814	59.4	557	40.6	0.002**	158	11.5	620	45.2	593	43.3	0.318
25 years and above	181	10.6	1,212	71.3	308	18.1		916	53.9	785	46.1		209	12.3	802	47.1	690	40.6	
**Education status**																			
Primary school	5	4.1	81	66.4	36	29.5	< 0.001***	56	45.9	66	54.1	0.001**	25	20.5	56	45.9	41	33.6	0.009**
High school	70	10.4	490	72.5	116	17.2		348	51.5	328	48.5		101	14.9	294	43.5	281	41.6	
Vocational School	69	14.0	384	77.9	40	8.1		276	56.0	217	44.0		56	11.4	229	46.5	208	42.2	
Undergraduate	255	16.4	1,127	72.4	174	11.2		910	58.5	646	41.5		164	10.5	733	47.1	659	42.4	
Graduate	35	15.6	149	66.2	41	18.2		140	62.2	85	37.8		21	9.3	110	48.9	94	41.8	
**Marital status**																			
Single	338	16.6	1,509	74.2	186	9.1	< 0.001***	1,185	58.3	848	41.7	0.001**	236	11.6	908	44.7	889	43.7	0.008**
Married	96	9.2	722	69.5	221	21.3		545	52.5	494	47.5		131	12.6	514	49.5	394	37.9	
**Income status**																			
Income < Expense	103	17.5	419	71.0	68	11.5	0.021*	340	57.6	250	42.4	0.039*	57	9.7	259	43.9	274	46.4	< 0.001***
Income = Expense	205	13.5	1,121	73.9	191	12.6		879	57.9	638	42.1		187	12.3	763	50.3	567	37.4	
Income > Expense	126	13.1	691	71.6	148	15.3		511	53.0	454	47.0		123	12.7	400	41.5	442	45.8	
**Smoking**																			
No	193	10.7	1,344	74.4	269	14.9	< 0.001***	925	51.2	881	48.8	< 0.001***	241	13.3	867	48.0	698	38.6	< 0.001***
Yes	241	19.0	887	70.1	138	10.9		805	63.6	461	36.4		126	10.0	555	43.8	585	46.2	
**Alcohol consumption**																			
No	305	12.5	1,788	73.5	338	13.9	< 0.001***	1,282	52.7	1,149	47.3	< 0.001***	284	11.7	1,166	48.0	981	40.4	0.001**
Yes	129	20.1	443	69.1	69	10.8		448	69.9	193	30.1		83	12.9	256	39.9	302	47.1	
**BMI groups**																			
Underweight	33	18.0	132	72.1	18	9.8	0.003**	91	49.7	92	50.3	0.130	30	16.4	97	53.0	56	30.6	0.021*
Normal	235	13.6	1,268	73.6	220	12.8		988	57.3	735	42.7		212	12.3	788	45.7	723	42.0	
Overweight	126	13.5	687	73.6	121	13.0		513	54.9	421	45.1		102	10.9	420	45.0	412	44.1	
Obesity	40	17.2	144	62.1	48	20.7		138	59.5	94	40.5		23	9.9	117	50.4	92	39.7	

Pearson chi-square;

*p < 0.05;

**p < 0.01;

***p < 0.001.

When comparing the chronotypes, sleep quality, and physical activity levels of participants, it is observed that individuals with an evening chronotype constitute 18.4% of those with poor sleep quality, while those with a morning chronotype have a higher percentage (19.7%) of normal sleep quality (*p* < 0.001). Additionally, individuals with an intermediate chronotype exhibit a higher level of physical activity (76.2%), and as inactivity increases, morning chronotype prevalence also rises (*p* = 0.003). Furthermore, individuals with normal sleep quality show a higher level of inactivity (14%) compared to those with poor sleep quality (10.3%), and conversely, individuals with high physical activity also exhibit a higher prevalence of poor sleep quality (*p* = 0.002) (Table 5).

**TABLE 5:** Comparison of participants’ chronotypes, sleep quality, and physical activity levels.

	Chronotype						
	Evening type		Intermediate type		Morning type		*p*
	*n*	%	*n*	%	*n*	%	
**PSQI**							
Poor	319	18.4	1,268	73.3	143	8.3	< 0.001**
Normal	115	8.6	963	71.8	264	19.7	
**MET**							
Inactive	51	13.9	257	70.0	59	16.1	0.003*
Minimally active	222	15.6	996	70.0	204	14.3	
Highly active	161	12.5	978	76.2	144	11.2	
**PSQI**							
Poor	179	10.3	794	45.9	757	43.8	0.002*
Normal	188	14.0	628	46.8	526	39.2	

Pearson chi-square;

*p < 0.01;

**p < 0.001.

Table 6 provides insight into the relationships between chronotype, physical activity (MET levels), and their combined effects on sleep quality (PSQI scores). The table is divided into two models.

**TABLE 6:** The effect of chronotype on physical activity and the effect of chronotype and physical activity on sleep quality.

		β	SE	OR (%95 CI)	*p*
**1 Model 1 (R^2^: 0.082)**					
	Constant	−1.481	0.201	0.227	< 0.001
	PSQI ← MET	−0.179	0.056	0.836 (0.750–0.932)	0.002*
	PSQI ← CHRONO	0.818	0.075	2.265 (1.954–2.626)	< 0.001*
**2 Model 2 (R^2^: 0.006)**					
**2.1**	**MET ← CHRONO (Inactive)** [ref.: highly active]				
	Intercept	−0.892	0.155		< 0.001
	Group 1	0.257	0.223	0.773 (0.499–1.197)	0.248
	Group 2	−0.444	0.170	0.641 (0.460–0.894)	0.009*
	Group 3	0			
**2.2**	**MET ← CHRONO (Minimal active)** [ref.: highly active]				
	Intercept	0.348	0.109		0.001
	Group 1	−0.027	0.150	0.973 (0.725–1.307)	0.857
	Group 2	0.330	0.118	0.719 (0.571–0.906)	0.005*
	Group 3	0			
**2.3**	**MET ← CHRONO (Minimal Active)** [ref.: inactive]				
	Intercept	1.241	0.148		< 0.001
	Group 1	0.230	0.214	1.259 (0.827–2.002)	0.248
	Group 2	0.114	0.164	1.121 (1.118–2.175)	0.485
	Group 3	0			

Coding: MET: inactive (1), minimal active (2), highly active (3); CHRONO: evening type (1), intermediate type (2), morning type (3); PSQI: poor (1), normal (2); Model 1: logistic regression; Model 2: multinomial logistic regression; OR: odds ratio; CI: confidence interval;

*p < 0.05.

In Model 1, a logistic regression model was employed to investigate the influence of chronotype and physical activity on sleep quality. This model yielded significant effects on the dependent variable, PSQI, for both independent variables, MET and CHRONO, explaining 8.2% of the variance. In particular, as MET levels transitioned from inactive to highly active, it was observed to increase the odds of moving from normal to poor PSQI categories by 16.4%. Conversely, shifting from an evening chronotype to a morning chronotype was associated with a substantial 126.5-fold increase in the odds of moving from poor to normal PSQI categories within the CHRONO variable.

Based on these findings, the logistic regression model and the equations representing the probabilities of experiencing normal or poor sleep can be expressed as follows:

$$\begin{array}{c} \text{ln}(\frac{P\left(PSQI=Normal\right)}{P\left(PSQI=Poor\right)}\\ =-1.481-0.179*\left(M\,E\,T\right)+0.818*\left(C\,H\,R\,O\,N\,O\right) \end{array}$$

$$\begin{array}{c} P\left(PSQI=Normal\right)=\\ \frac{e^{-1.481-0.179*\left(M\,E\,T\right)+0.818*\left(C\,H\,R\,O\,N\,O\right)}}{1+e^{-1.481-0.179*\left(M\,E\,T\right)+0.818*\left(C\,H\,R\,O\,N\,O\right)}} \end{array}$$

P(PSQI=Poor)=1-P(PSQI=Normal)

In Model 2, the influence of chronotype on physical activity was examined using multinomial logistic regression. The overall model (Model 2) was found to explain only 0.6% of the total variance. Within Model 2.1 and Model 2.2, the reference category was set as “highly active” compared to the MET group, while in Model 2.3, it was defined as “inactive.” Within the CHRONO group, the reference category was “morning chronotype.”

In Model 2.1, an evaluation was conducted within the inactive group in reference to the highly active group. It was observed that, compared to a highly active individual, an inactive individual had a significant effect on being of intermediate chronotype, reducing the probability by 35.9%. However, the probability of being an evening chronotype compared to a morning chronotype for an inactive individual as opposed to a highly active individual was not significant.

In Model 2.2, an evaluation was performed within the minimally active group in reference to the highly active group. It was found that, compared to a highly active individual, a minimally active individual had a significant effect on being of intermediate chronotype, reducing the probability by 28.1%. However, the probability of being an evening chronotype compared to a morning chronotype for a minimally active individual as opposed to a highly active individual was not significant.

In Model 2.3, an evaluation was conducted within the minimally active group in reference to the inactive group. It was found that the probability of being an evening chronotype or an intermediate chronotype compared to a morning chronotype for a minimally active individual as opposed to an inactive individual was not significant.

Based on these findings, the significant models of multinomial regression can be expressed as follows.

$$\begin{array}{c} \text{ln}(\frac{P\left(MET=Inactive\right)}{P\left(MET=HighlyActive\right)}=\\ -0.892-0.444*\left(I\,n\,t\,e\,r\,m\,e\,d\,i\,a\,t\,e\,T\,y\,p\,e\right)+0.257*\left(E\,v\,e\,n\,i\,n\,g\,T\,y\,p\,e\right) \end{array}$$

$$\begin{array}{c} \text{ln}(\frac{P\left(MET=Minimalactive\right)}{P\left(MET=HighlyActive\right)}=\\ 0.348-0.027*\left(E\,v\,e\,n\,i\,n\,g\,T\,y\,p\,e\right)+0.330*\left(I\,n\,t\,e\,r\,m\,e\,d\,i\,a\,t\,e\,T\,y\,p\,e\right) \end{array}$$

For the probabilities of being inactive, minimally active, and highly active within the significant models of regression, the equations are as follows:

$$\begin{array}{c} P\left(MET=Inactive\right)=\\ \frac{e^{-0.892+0.257*\left(E\,v\,e\,n\,i\,n\,g\,T\,y\,p\,e\right)-0.444*\left(I\,n\,t\,e\,r\,m\,e\,d\,i\,a\,t\,e\,T\,y\,p\,e\right)}}{\begin{array}{c} 1+e^{-0.892+0.257*\left(E\,v\,e\,n\,i\,n\,g\,T\,y\,p\,e\right)-0.444*\left(I\,n\,t\,e\,r\,m\,e\,d\,i\,a\,t\,e\,T\,y\,p\,e\right)}+\\ e^{0.348-0.027*\left(E\,v\,e\,n\,i\,n\,g\,T\,y\,p\,e\right)+0.330*\left(I\,n\,t\,e\,r\,m\,e\,d\,i\,a\,t\,e\,T\,y\,p\,e\right)} \end{array}} \end{array}$$

$$\begin{array}{c} P\left(MET=MinimalActive\right)=\\ \frac{e^{-0.892+0.257*\left(E\,v\,e\,n\,i\,n\,g\,T\,y\,p\,e\right)-0.444*\left(I\,n\,t\,e\,r\,m\,e\,d\,i\,a\,t\,e\,T\,y\,p\,e\right)}}{\begin{array}{c} 1+e^{-0.892+0.257*\left(E\,v\,e\,n\,i\,n\,g\,T\,y\,p\,e\right)-0.444*\left(I\,n\,t\,e\,r\,m\,e\,d\,i\,a\,t\,e\,T\,y\,p\,e\right)}+\\ e^{0.348-0.027*\left(E\,v\,e\,n\,i\,n\,g\,T\,y\,p\,e\right)+0.330*\left(I\,n\,t\,e\,r\,m\,e\,d\,i\,a\,t\,e\,T\,y\,p\,e\right)} \end{array}} \end{array}$$

$$\begin{array}{c} P\left(MET=HighlyActive\right)=\\ 1-\left[P\left(MET=Inactive\right)+P\left(MET=MinimalActive\right)\right] \end{array}$$

Table 7 reveals significant differences in dietary intake based on participants’ chronotypes. Male individuals with a morning chronotype have a significantly higher energy intake (kcal) compared to those with intermediate and evening chronotypes (*p* = 0.011). Among females, those with an intermediate chronotype consume significantly more carbohydrates (g) than morning types (*p* = 0.006). For protein intake (g), both intermediate and morning chronotypes in females have significantly higher consumption levels than the evening types (*p* = 0.001), with a similar but non-significant trend in males (*p* = 0.069). Regarding the percentage of protein intake, females with an evening chronotype consume significantly less compared to morning and intermediate types (*p* = 0.001). Males with intermediate and morning chronotypes have significantly higher total fat intake (g and %) compared to those with an evening chronotype (*p* < 0.001). For specific fatty acids (SFA, MUFA, and PUFA), a similar trend is observed. Female participants classified as intermediate and morning types have significantly higher cholesterol intake than those with an evening chronotype (*p* = 0.001). Lastly, males with a morning chronotype consume significantly more dietary fiber compared to intermediate and evening types (*p* = 0.001).

**TABLE 7:** Comparison of participants’ chronotypes and dietary intakes.

		Chronotype				
		Mean ± SD Q_2_ (Q_1_-Q_3_)			*p*	*Post-hoc*
	Sex	Evening type	Intermediate type	Morning type		
Energy (kcal)	Female	2,558.19 ± 850.15	2,621 ± 779.3	2,641.16 ± 828.38	0.453_a_	
		2,573.69 (1,846.6–3,088.97)	2,635.17 (2,073.93–3,129.1)	2,635.17 (2,028.13–3,230.59)		
	Male	2,702.49 ± 850.86	2,744.71 ± 874.43	2,935.57 ± 788.38	**0.011** * _a_	3 > 1, 2
		2,674.26 (2,061.17–3,410.72)	2,730.06 (2,091.9–3,376.02)	2,965.56 (2,391.37–3,549.38)		
Carbohydrate (g)	Female	230.2 ± 76.61	241.03 ± 77.06	225.33 ± 79.54	**0.006** ** _a_	2 > 3
		230.1 (170.51–282.06)	239.37 (189.54–294.44)	232.16 (169.8–274.86)		
	Male	251.22 ± 85.98	251.52 ± 88.05	255.39 ± 77.12	0.876_a_	
		253.19 (197.35–322.25)	250.65 (185.73–315.63)	251 (207.29–306.7)		
Carbohydrate (%)	Female	0.37 ± 0.09	0.37 ± 0.09	0.34 ± 0.08	0.115_a_	
		0.36 (0.31–0.43)	0.36 (0.32–0.43)	0.34 (0.28–0.4)		
	Male	0.38 ± 0.1	0.37 ± 0.09	0.35 ± 0.07	**0.005** ** _k_	1, 2 > 3
		0.37 (0.31–0.43)	0.36 (0.31–0.42)	0.35 (0.31–0.38)		
Protein (g)	Female	88.93 ± 33.49	95.93 ± 31.18	100.33 ± 31.13	**0.001** **_a_	2, 3 > 1
		88.85 (64.09–108.98)	96.02 (73.68–114.96)	100.1 (76.63–122.17)		
	Male	105.02 ± 32.75	104.75 ± 34.83	111.09 ± 32.32	0.069_a_	
		105.27 (82.53–127.91)	101.6 (80.42–127.44)	109.68 (91.38–131.36)		
Protein (%)	Female	0.14 ± 0.04	0.15 ± 0.04	0.16 ± 0.04	**0.001** ** _k_	3 > 1, 2 2 > 1
		0.14 (0.12–0.16)	0.15 (0.13–0.17)	0.15 (0.14–0.17)		
	Male	0.16 ± 0.04	0.16 ± 0.04	0.15 ± 0.03	0.138_k_	
		0.15 (0.14–0.18)	0.15 (0.13–0.18)	0.15 (0.13–0.17)		
Fat (g)	Female	143.04 ± 60.5	142.24 ± 55.71	149.56 ± 57.25	0.307_a_	
		143.53 (92.74–192.4)	143.53 (97.26–184.82)	148.19 (104.97–192.94)		
	Male	142.54 ± 60.49	147.32 ± 58.58	164.11 ± 54.39	**< 0.001** ***_a_	2, 3 > 1
		142.95 (94.63–187.97)	145.35 (101.09–191.46)	163.64 (130.76–207.26)		
Fat (%)	Female	0.49 ± 0.1	0.48 ± 0.1	0.5 ± 0.09	**0.001** **_a_	3 > 2
		0.5 (0.41–0.56)	0.49 (0.4–0.55)	0.51 (0.44–0.57)		
	Male	0.46 ± 0.11	0.47 ± 0.1	0.49 ± 0.08	**0.001** **_a_	2, 3 > 1
		0.47 (0.39–0.53)	0.49 (0.4–0.54)	0.49 (0.45–0.54)		
SFA (g)	Female	39.78 ± 15.85	40.63 ± 14.1	42.54 ± 14.78	0.150_a_	
		41.06 (27.93–50.68)	40.66 (30.99–50.13)	41.06 (31.66–54.41)		
	Male	41.78 ± 15.22	42.53 ± 15.33	46.52 ± 14.79	**0.002** **_a_	2, 3 > 1
		42.08 (30.91–53.08)	41.76 (31.38–53.67)	46.72 (36.96–59.38)		
MUFA (g)	Female	66.26 ± 34.39	64.95 ± 32.19	69.57 ± 31.71	0.310_k_	
		69.19 (35.01–95.95)	65.29 (37.34–92.05)	73.78 (42.15–95.73)		
	Male	63.06 ± 33.76	66.54 ± 32.39	74.59 ± 30.19	**0.001** ** _k_	3 > 1, 2
		62.17 (32.08–91.15)	65.29 (38.51–94.53)	76.25 (53.34–99.72)		
PUFA (g)	Female	28.79 ± 11.66	28.3 ± 11.73	28.63 ± 12.45	0.894_a_	
		28.53 (19.55–37.52)	28.24 (19.17–36.76)	27.44 (19.22–38.1)		
	Male	28.94 ± 13.04	29.38 ± 12.86	33.37 ± 11.8	**< 0.001** ***_a_	2, 3 > 1
		28.31 (19.11–38.57)	28.67 (19.3–39.47)	33.39 (24.22–44.07)		
Cholesterol (mg)	Female	412.48 ± 232.61	460.18 ± 224.15	494.15 ± 201.99	**0.001** **_a_	2, 3 > 1
		396.88 (258.91–536.26)	424.68 (327.83–603.58)	495.4 (367.91–622.13)		
	Male	564.66 ± 270.96	560.94 ± 256.67	581.55 ± 232.19	0.635_*a*_	
		588.18 (386.52–751.26)	551.89 (383.03–757.68)	572.99 (412.98–742.33)		
Fiber (g)	Female	27.66 ± 10.95	29.06 ± 10.91	29.29 ± 11.38	0.172_*a*_	
		27.31 (19.67–34.92)	28.67 (20.82–36.65)	28.82 (21.28–37.15)		
	Male	28.83 ± 11.75	29.6 ± 12.29	32.89 ± 10.96	**0.001** **_a_	3 > 1, 2
		28.89 (20.23–36.21)	28.85 (19.58–38.5)	33.76 (24.61–40.43)		

^*a*^One-way ANOVA.

^*k*^Kruskal–Wallis test.

*p < 0.05;

**p < 0.01;

***p < 0.001. SFA, saturated fatty acids; MUFA, monounsaturated fatty acids; PUFA, polyunsaturated fatty acids.

The analysis of dietary habits concerning sleep quality reveals noteworthy differences (Table 8). Male participants with normal sleep quality display significantly lower energy intake (kcal) compared to those with poor sleep quality (*p* < 0.001), a trend similarly observed in females (*p* = 0.001). Additionally, both male and female individuals with normal sleep quality consume significantly fewer carbohydrates (g) than those with poor sleep quality (*p* < 0.001). While there are no significant differences in carbohydrate percentage between sleep quality groups, there are significant disparities in protein intake (g). Females with poor sleep quality consume more protein than those with normal sleep quality (*p* = 0.002), and a similar trend is observed in males (*p* < 0.001). In terms of dietary fat intake (g), males with normal sleep quality have lower consumption levels compared to those with poor sleep quality (*p* = 0.033), whereas there are no significant differences among females. However, the percentage of dietary fat varies, with males displaying lower fat percentages with normal sleep quality (*p* = 0.014). Furthermore, participants with poor sleep quality consume significantly higher levels of SFA (females: *p* = 0.026; males: *p* = 0.002) and PUFA (*p* < 0.001, both) in comparison to those with normal sleep quality. Cholesterol intake is also significantly higher in participants with poor sleep quality (females: *p* = 0.007; males: *p* < 0.001). Lastly, dietary fiber intake is notably lower in both male and female individuals with normal sleep quality (*p* < 0.001).

**TABLE 8:** Comparison of participants’ sleep quality and dietary intakes.

		Sleep quality		
		Mean ± SD Q_2_ (Q_1_-Q_3_)		*p*
	Sex	Poor	Normal	
Energy (kcal)	Female	2,667.28 ± 817.51	2,535.54 ± 756.79	**0.001** **_t_
		2,636.76 (2,070.29–3,237.67)	2,538.05 (2,021.91–2,996.85)	
	Male	2,863.26 ± 890.21	2,656.36 ± 817.62	**< 0.001** ***_t_
		2,869.31 (2,238.01–3,544.84)	2,635.17 (2,059.18–3,251.36)	
Carbohydrate (g)	Female	244 ± 80.59	227.65 ± 71.61	**< 0.001** ***_t_
		239.37 (187.42–300.01)	230.2 (180.46–273.15)	
	Male	263.84 ± 90.56	238.96 ± 79.45	**< 0.001** ***_t_
		263.57 (199.28–334.57)	239.37 (183.65–295.22)	
Carbohydrate (%)	Female	0.37 ± 0.09	0.37 ± 0.09	0.234_t_
		0.36 (0.31–0.43)	0.36 (0.31–0.42)	
	Male	0.37 ± 0.08	0.37 ± 0.09	**0.006** **_u_
		0.36 (0.32–0.43)	0.36 (0.31–0.41)	
Protein (g)	Female	97.53 ± 32.99	92.47 ± 29.26	**0.002** **_t_
		98.08 (74.32–118.08)	93.19 (72.07–111.27)	
	Male	111.33 ± 35.07	99.4 ± 32.26	**< 0.001** ***_t_
		109.86 (86.54–136.4)	98.08 (78.8–120.07)	
Protein (%)	Female	0.15 ± 0.04	0.15 ± 0.03	0.445_u_
		0.15 (0.13–0.17)	0.15 (0.13–0.17)	
	Male	0.16 ± 0.04	0.15 ± 0.04	**< 0.001** ***_u_
		0.15 (0.14–0.18)	0.15 (0.13–0.17)	
Fat (g)	Female	145.23 ± 57.58	140.37 ± 55.12	0.098_t_
		143.53 (99.28–189.26)	143.53 (95.42–182.04)	
	Male	151.98 ± 59.56	145.6 ± 57.41	**0.033** *_t_
		148.19 (106.07–201.16)	148.49 (99.2–187.25)	
Fat (%)	Female	0.48 ± 0.1	0.48 ± 0.1	0.220_t_
		0.49 (0.41–0.55)	0.49 (0.41–0.56)	
	Male	0.47 ± 0.09	0.48 ± 0.1	**0.014** *_t_
		0.48 (0.4–0.53)	0.49 (0.42–0.55)	
SFA (g)	Female	41.42 ± 14.59	39.75 ± 14.22	**0.026** *_t_
		41.06 (31.02–52.03)	39.67 (30.33–49.49)	
	Male	44.13 ± 15.53	41.69 ± 14.96	**0.002** **_t_
		43.65 (32.49–55.98)	41.06 (31.1–52.92)	
MUFA (g)	Female	66.03 ± 33	65.28 ± 31.7	0.301_u_
		65.29 (37.81–94.64)	66.89 (37.31–91.54)	
	Male	67.31 ± 32.5	66.97 ± 32.4	0.421_u_
		65.29 (40.53–95.54)	67.39 (37.32–94.62)	
PUFA (g)	Female	29.28 ± 11.99	27.1 ± 11.43	**< 0.001** ***_t_
		28.67 (19.88–37.86)	25.53 (18.18–35.34)	
	Male	31.37 ± 13.04	28.2 ± 12.38	**< 0.001** ***_t_
		31.5 (21.59–42.61)	27.39 (18.49–38.05)	
Cholesterol (mg)	Female	470.14 ± 232.67	439.26 ± 208.06	**0.007** **_t_
		442.29 (326.52–620.32)	414.29 (326.59–562.45)	
	Male	600.98 ± 261.03	523.78 ± 243.19	**< 0.001** ***_t_
		614.51 (407.8–826.03)	498.69 (364.51–675.65)	
Fiber (g)	Female	29.75 ± 11.24	27.6 ± 10.46	**< 0.001** ***_t_
		28.82 (21.99–37.14)	26.46 (19.83–35.16)	
	Male	31.74 ± 12.55	27.95 ± 11.25	**< 0.001** ***_t_
		32.48 (22.19–42.02)	27.5 (19.01–36.57)	

^*t*^Tukey’s HSD test. ^*u*^Mann–Whitney U test.

*p < 0.05;

**p < 0.01;

***p < 0.001. SFA, saturated fatty acids; MUFA, monounsaturated fatty acids; PUFA, polyunsaturated fatty acids.

Table 9 provides the relationship between physical activity levels and dietary parameters, categorized by sex. The results reveal significant variations in dietary parameters across different levels of physical activity and between sexes. In the context of energy intake (kcal), females exhibited statistically significant differences (*p* = 0.012) among groups. *Post-hoc* analysis indicated that the inactive group consumed significantly less energy than more active groups. In males, the difference in energy intake was even more pronounced (*p* < 0.001), with the highly active group consuming significantly more energy than both the minimally active and inactive groups. Similar patterns of significance were observed in carbohydrate, protein, and fat intake (g and %) for both sexes, highlighting the influence of physical activity on dietary choices. Contrary to other macronutrient intakes fat percentages were higher in minimally active females than highly active ones (*p* = 0.004) and inactive males than highly active ones (*p* = 0.027). Additionally, highly active males had significantly higher cholesterol intake than other groups, while highly active females had significantly higher cholesterol intake compared to minimally active females (*p* < 0.001, both). Furthermore, fiber intake showed significant differences, with the highly active group consuming more fiber than the other two groups in both sexes (females: *p* = 0.004; males: *p* < 0.001).

**TABLE 9:** Comparison of participants’ physical activity and dietary intakes.

		Physical activity				
		Mean ± SD Q_2_ (Q_1_-Q_3_)			*p*	*Post-hoc*
	Sex	Inactive	Minimal active	Highly active		
Energy (kcal)	Female	2,449.58 ± 816.88	2,633.9 ± 786.39	2,636.11 ± 798.62	**0.012** *_a_	2, 3 > 1
		2,586.79 (1,809.43–3,013.51)	2,635.17 (2,094.52–3,168.29)	2,635.17 (2,040.14–3,228.26)		
	Male	2,566.19 ± 962.52	2,731.93 ± 871.67	2,851.34 ± 811.31	**< 0.001** ***_a_	3 > 1, 2 2 > 1
		2,531.05 (1,856.27–3,295.17)	2,703.38 (2,121.45–3,368.55)	2,851.59 (2,271.16–3,431.34)		
Carbohydrate (g)	Female	214.78 ± 74.42	234.97 ± 74.1	247.43 ± 81.28	**< 0.001** ***_a_	3 > 1, 2 2 > 1
		222.6 (161.78–262.11)	237.07 (180.76–290.39)	239.37 (194.57–298.8)		
	Male	226.24 ± 96.18	246.31 ± 86.28	264.49 ± 81.12	**< 0.001** ***_a_	3 > 1, 2 2 > 1
		235.55 (140.57–299.27)	245.5 (180.77–308.01)	261.59 (207.94–323.17)		
Carbohydrate (%)	Female	0.36 ± 0.1	0.36 ± 0.08	0.38 ± 0.09	**0.004** **_a_	3 > 2
		0.36 (0.3–0.41)	0.36 (0.31–0.41)	0.37 (0.32–0.44)		
	Male	0.35 ± 0.1	0.37 ± 0.09	0.38 ± 0.09	**0.002** **_k_	3 > 1, 2
		0.35 (0.3–0.4)	0.36 (0.31–0.41)	0.36 (0.32–0.43)		
Protein (g)	Female	90.34 ± 34.57	94.67 ± 30.21	98.14 ± 32.44	**0.009** **_a_	3 > 1
		97.59 (66.76–112.06)	96.01 (73.04–114.11)	95.74 (74.58–120.2)		
	Male	96.58 ± 36.93	103.79 ± 34.27	109.93 ± 32.8	**< 0.001** ***_a_	3 > 1, 2 2 > 1
		98.08 (74.87–120.1)	103.13 (77.78–126.84)	105.88 (87.65–131.83)		
Protein (%)	Female	0.15 ± 0.04	0.15 ± 0.04	0.15 ± 0.04	**0.018** *_k_	3 > 2
		0.15 (0.12–0.17)	0.15 (0.12–0.17)	0.15 (0.13–0.17)		
	Male	0.15 ± 0.05	0.16 ± 0.04	0.16 ± 0.03	0.391_k_	
		0.15 (0.13–0.18)	0.15 (0.13–0.18)	0.15 (0.13–0.18)		
Fat (g)	Female	137.24 ± 56.67	146.97 ± 56.56	140.03 ± 56.46	**0.023** *_a_	2 > 1
		142.4 (96.59–176.13)	147.99 (103.6–188.99)	141.45 (92.91–183.19)		
	Male	142.3 ± 61.46	148.66 ± 58.04	151.1 ± 58.18	0.142_a_	
		143.53 (93.09–191.16)	151.4 (106.27–191.89)	147.95 (103.67–197.31)		
Fat (%)	Female	0.49 ± 0.11	0.49 ± 0.1	0.47 ± 0.1	**0.004** **_a_	2 > 3
		0.49 (0.43–0.56)	0.5 (0.42–0.56)	0.47 (0.39–0.54)		
	Male	0.49 ± 0.11	0.48 ± 0.09	0.46 ± 0.1	**0.027** *_a_	1 > 3
		0.49 (0.42–0.56)	0.49 (0.42–0.54)	0.48 (0.39–0.54)		
SFA (g)	Female	39.16 ± 15.24	41.48 ± 14.48	40.22 ± 14.17	0.072_a_	
		41.06 (28.92–48.56)	41.06 (31.3–51.58)	39.59 (30.4–49.82)		
	Male	40.2 ± 16.87	42.88 ± 15.36	43.84 ± 14.69	**0.009** **_a_	3 > 1
		41.06 (26.1–53.24)	42.69 (31.67–54.21)	42.75 (32.81–54.84)		
MUFA (g)	Female	62.83 ± 31.42	68.31 ± 32.54	63.05 ± 32.44	**0.008** **_k_	2 > 1, 3
		65.29 (37.49–87.48)	71.75 (40.67–93.94)	60.9 (34.66–91.65)		
	Male	64.61 ± 32.85	67.1 ± 31.6	67.93 ± 33.04	0.038_k_	
		65.36 (34.54–91.71)	69.94 (39.35–94.06)	64.99 (39.75–97.04)		
PUFA (g)	Female	27.15 ± 11.82	28.64 ± 11.73	28.46 ± 11.92	0.276_a_	
		28.38 (18.36–34.37)	28.39 (19.56–37.63)	27.78 (18.97–37.26)		
	Male	29.08 ± 13.35	29.77 ± 13.08	30.17 ± 12.43	0.503_a_	
		28.67 (18.45–41.21)	29.26 (19.05–39.95)	28.94 (20.47–40.05)		
Cholesterol (mg)	Female	446.83 ± 221.31	438.36 ± 210.79	487.49 ± 237.8	**< 0.001** ***_a_	3 > 2
		446.78 (315.19–595.31)	410.64 (325.33–565.56)	455.91 (333.74–652.3)		
	Male	518.58 ± 260.78	549 ± 244.15	591.03 ± 261.14	**< 0.001** ***_a_	3 > 1, 2 2 > 1
		541.2 (321.2–691.1)	547.48 (383.41–715.94)	577.4 (395.88–815.7)		
Fiber (g)	Female	26.55 ± 11.01	28.82 ± 10.87	29.66 ± 11.04	**0.004** **_a_	2, 3 > 1
		27.79 (17.46–32.72)	28.34 (20.5–36.78)	28.72 (21.97–36.9)		
	Male	27.63 ± 12.95	29.2 ± 12.31	31.27 ± 11.48	**< 0.001** ***_a_	3 > 1, 2
		28.21 (16.54–37.17)	28.75 (19.68–38.18)	30.8 (22.69–40.02)		

^*a*^One-way ANOVA ^*k*^Kruskal–Wallis test;

*p < 0.05;

**p < 0.01;

***p < 0.001. SFA: saturated fatty acids; MUFA: monounsaturated fatty acids; PUFA: polyunsaturated fatty acids.

## 4 Discussion

The present study delves into the intricate interplay of chronotype, sleep quality, physical activity, and dietary intake, aiming to contribute substantively to our understanding of the relationships between these pivotal domains within a diverse participant cohort.

### 4.1 Chronotype and sleep quality

The analysis revealed significant associations between chronotype and sleep quality. Individuals with an evening chronotype were more likely to experience poor sleep quality. This finding aligns with existing literature, where evening chronotypes are often associated with delayed sleep onset, shorter sleep duration, and higher rates of sleep disturbances (7, 23–25). The propensity for evening chronotypes to encounter poor sleep quality may stem from societal schedules that demand early wake times, creating a misalignment between their natural sleep-wake patterns and external demands. In contrast, morning chronotypes align more closely with conventional schedules, promoting better sleep quality. A cross-sectional study conducted with the participation of 5,497 medical students found being an evening type was the strongest predictor of poor sleep quality, underscoring the disharmony between real-life demands of studying medicine and evening chronotype and suggesting a shift toward a morning chronotype for better sleep (8). This association underscores the importance of considering an individual’s chronotype when evaluating sleep quality and designing interventions to address sleep-related issues.

### 4.2 Physical activity and sleep quality

The relationship between physical activity and sleep quality in our study underscores the potential benefits of mild to moderate exercise in promoting restful sleep. Highly active individuals were more likely to experience poor sleep quality. This finding may be attributed to the stimulating effects of vigorous physical activity close to bedtime, which can disrupt the natural transition into sleep. Dubinina et al. (26) suggest high physical activity load at work or frequent vigorous physical activity is associated with difficulties initiating sleep and may be a risk factor for insomnia. Another study presents the relationship between increased physical activity and decreased sleep time in adolescents (27). On the other hand, a meta-analysis showed moderate to high-intensity physical activity was associated with better sleep quality, even though the majority of the included studies did not find any association (9). Although there is no consensus on the intense part, many studies revealed the beneficial effects of physical activity for better sleep (3, 28).

### 4.3 Chronotype and physical activity

The findings of this study revealed significant associations between chronotype and physical activity level. It was observed that individuals with an evening chronotype were more likely to be minimally active. Furthermore, as physical inactivity increased, the prevalence of morning chronotype also rose. These findings are incongruent with existing literature, which usually established a correlation between being a morning type and being highly active and vice-versa, an evening type with inactive (1, 29, 30). However, a systematic review of chronotype, physical activity, and sports performance suggests evening types are less active and perform less in the morning than intermediate and morning types. They conclude that the chronotype effect on physical activity is not consistent (31). These findings suggest a complex relationship between chronotype and physical activity, wherein further investigations that delve into the causality are imperative to elucidate the intricate mechanisms at play and enhance our comprehensive understanding of this multifaceted topic.

### 4.4 Chronotype and dietary intake

The study suggested intriguing associations between dietary intake and chronotype. Male individuals with a morning chronotype exhibited significantly higher energy intake, while females with an intermediate chronotype consumed more carbohydrates. Furthermore, both male and female participants with an evening chronotype reported lower protein intake percentages. These findings align with the previous studies. A study conducted with 112 young Japanese women suggests evening chronotype individuals’ energy, protein, and cholesterol intakes were lower (32). Toktas et al. (33) compared 12 morning-type and 11 evening-type male university students’ dietary intakes and found those with an evening chronotype tendency consume more energy, carbohydrates, and total fat and lower protein. Also, several systematic reviews pointed out that the evening chronotype is associated with poorer dietary habits (1, 2, 34). Nevertheless, there are studies with minor distinctions. Arslan et al. (35) investigated the chronotype and nutritional status of 204 healthcare professionals and found carbohydrate intakes were higher in evening types. Similarly, Bodur et al. (23) found carbohydrate and energy intakes were higher, with no other significant differences in terms of chronotype and nutrients. These findings suggest that chronotype may influence dietary choices, potentially due to variations in meal timing and food preferences associated with different chronotypes. Morning chronotypes may have more substantial breakfast consumption, contributing to higher energy intake, while evening chronotypes may prefer late-night snacking or delayed meal patterns, affecting their macronutrient distribution. These associations underscore the importance of considering chronotype when assessing dietary habits and developing personalized nutritional recommendations.

### 4.5 Sleep quality and dietary intake

The analysis also revealed significant relationships between sleep quality and dietary intake. Participants with normal sleep quality reported lower energy intake and consumed fewer carbohydrates in both sexes. Additionally, individuals with normal sleep quality had lower total fat intake in males. Poor sleep quality was associated with higher protein intake in both sexes, as well as increased intake of SFA, PUFA, cholesterol, and fiber. Another large-scale cross-sectional study conducted in Türkiye with 2,446 participants found similar energy and macronutrient intakes across poor and normal sleep quality categories, except for fiber intakes were higher in those who had normal sleep quality. However, they also stated individuals with shorter sleep periods had higher SFA intakes (13). Agostini et al. (36) found that poor sleep quality is associated with poor dietary habits. They stated individuals with poor sleep quality tend to be missing breakfast and having energy-dense junk foods may contribute to higher energy and macronutrient consumption (36). Also, a meta-analysis that included intervention studies, concluded poor sleep quality, and sleeping less than 5.5 h increase energy and macronutrient intake (4). Another review supports these results stating poor sleep quality is associated with increased caloric consumption, poor dietary habits, and obesity (14). These findings indicate that sleep quality may exert a substantial influence on dietary choices. Individuals with poor sleep quality may exhibit alterations in appetite-regulating hormones, leading to increased food intake, particularly high-protein and high-fat foods, as observed in this study. Moreover, the association between poor sleep quality and higher total fat and SFA consumption may have implications for cardiovascular health, as elevated SFA intake is linked to adverse outcomes. Therefore, addressing sleep quality may be a key component of interventions aimed at improving dietary habits and overall health.

### 4.6 Physical activity and dietary intake

The study demonstrated significant variations in dietary parameters based on physical activity levels. Highly active individuals consumed more energy, carbohydrates, protein, and dietary fiber, reflecting increased energy expenditure and nutrient requirements associated with physical activity. In contrast, inactive individuals, particularly males, exhibited lower energy intake. These findings are expected and align with the literature (37, 38). However, the percentage of dietary fat was higher in minimally active females and inactive males compared to their highly active counterparts. A study found similar results; sedentary activities are positively associated with dietary fat percentages (39). The reason for this result may be that people with high physical activity may consciously stay away from high-fat foods. This variation in fat consumption may have implications for body composition and metabolic health and needs further investigation.

### 4.7 Implications for tailored interventions

The multidirectional interactions among chronotype, sleep quality, physical activity, and dietary intake underscore the complexity of health behaviors and their interplay. Understanding these relationships has significant implications for tailored interventions aimed at optimizing sleep, physical activity, and dietary behaviors to enhance overall wellbeing. Personalized strategies that consider an individual’s chronotype, physical activity level, and sleep quality can lead to more effective interventions that address specific needs and challenges. For example, interventions for evening chronotypes may focus on improving sleep hygiene and facilitating physical activity during preferred times, while morning chronotypes may benefit from dietary recommendations that align with their early eating patterns. Moreover, addressing the interrelationships between these factors can provide a holistic framework for promoting individual wellbeing.

## 5 Conclusion

This study contributes to the evolving body of knowledge elucidating the intricate interplay of chronotype, sleep quality, physical activity, and dietary intake. The findings underscore the need for personalized and multidimensional approaches to health promotion and intervention. Tailoring strategies to individual characteristics and considering the complex interactions between these factors can facilitate the development of holistic frameworks for enhancing wellbeing and optimizing health behaviors. Further research is warranted to deepen our understanding of these relationships and their implications for public health and clinical practice.

### 5.1 Limitations and future directions

While this study has provided valuable insights, it is essential to acknowledge its inherent limitations. The cross-sectional design employed in this research precludes the establishment of causal relationships between variables. As such, while associations were observed, definitive causation cannot be inferred. Longitudinal studies or intervention-based research would offer a more robust platform for establishing causal pathways between chronotype, sleep quality, physical activity, and dietary intake. Moreover, the reliance on self-reported data for dietary intake and physical activity introduces potential biases. Recall bias and social desirability bias are inherent risks associated with self-reported measures. Participants may tend to overestimate or underestimate their actual behaviors, affecting the accuracy of the collected data. Employing complementary methods, such as objective measures (e.g., accelerometers for physical activity, dietary logs, or biomarkers for dietary intake), could strengthen future investigations and provide a more comprehensive understanding of these health behaviors. Additionally, future research endeavors might benefit from delving deeper into the role of psychological factors. Exploring motivational aspects, self-regulation mechanisms, and individual differences in behavior change strategies could elucidate the underlying mechanisms influencing health behaviors. Understanding how these psychological factors mediate the relationships between chronotype, sleep quality, physical activity, and dietary intake could provide invaluable insights into tailoring interventions effectively. Acknowledging and addressing these limitations can guide future research to achieve a more comprehensive understanding of the complexities involved in health behaviors.

## Data availability statement

The raw data supporting the conclusions of this article will be made available by the author, without undue reservation.

## Ethics statement

The studies involving humans were approved by the Ethics Committee of İstanbul Okan University. The studies were conducted in accordance with the local legislation and institutional requirements. The participants provided their written informed consent to participate in this study.

## Author contributions

AG: Writing – original draft, Writing – review & editing.
